# Comparison of the teaching clinical biochemistry in face-to-face and the flex-flipped classroom to medical and dental students: a quasi-experimental study from IRAN

**DOI:** 10.1186/s12909-024-05051-8

**Published:** 2024-02-13

**Authors:** Zahra Karimian, Pooneh Mokarram, Nahid Zarifsanaiey

**Affiliations:** 1 Department of E-Learning in Medical Sciences, Virtual School and Center of Excellence in E-Learning, Shiraz University of Medical Sciences, Shiraz, Iran; 2 Autophagy Research Center, Department of Biochemistry, School of Medicine, Shiraz University of Medical Sciences, Zand Street, Shiraz, Iran

**Keywords:** Face-to-face education, e-learning, Blended learning, Flipped class, Flex model, Knowledge, Quality

## Abstract

**Introduction:**

Biochemistry is one of the main courses of basic sciences in the medical curriculum, along with other difficult subjects that are difficult to learn. The emergence of new technologies has made it possible to test new methods such as e-Learning. In this study, we compared two methods of Flex-Flipped Classroom (FFC) and face-to-face.

**Method:**

A quasi-experimental research was done which involved both medical and dental students studying the clinical biochemistry course in the joint semester in 2019. A total of 100 medical students were trained in biochemistry through face-to-face teaching, and 60 dental students were trained in the same course through the FFC model. Three researcher-made tools were used to compare the two groups to assess the student’s satisfaction, scores, and self-evaluation. The content validity of the tools was checked using the opinions of 10 experts through the CVI index. The results were analyzed using one-sample t-tests, independent t-tests, and ANOVA.

**Results:**

Both groups scored significantly more than the cut-off-point (Mean > 3.5) in their average scores of the total and sub-components of the self-evaluation questionnaire (*P* < 0.05). Face-to-face teaching was viewed more favorably than the FFC teaching except for considering the flexibility (4.14 ± 1.55), but the difference was not significant (*P* > 0.05). The students’ knowledge score in the FFC was slightly higher than that in the face-to-face method, but this difference was not significant(*P* = 0.758).

**Conclusion:**

Both face-to-face and FFC methods were effective according to the students, but the level of satisfaction with the face-to-face method was higher. It seems that teacher-student interaction is an important factor in students' preferences. However, the students preferred the flexibility of multimedia. It seems necessary to use the advantages of each method in a model appropriate to the students' conditions and available facilities.

## Introduction

Biochemistry is one of the main subjects of the basic sciences in the medical curriculum, which is important for understanding the clinical sciences of the medical profession [1, 2]. Meanwhile, it is one of the hardest courses in basic medicine and an abstract subject that is difficult for students to learn [3, 4]. The course content is full of biochemical structures, routes, formulas, materials, and metabolism [5]. The curriculum for biochemistry is very broad; the time the subject is usually taught is limited, and there are usually a large number of students in a class [6–9]. In this context, in most medical schools around the world, lecturers are forced to choose a lecture style that is often the best way to present a large amount of information to a large number of students [6, 8]. However, the one-sidedness and teacher-centeredness of the lecture method diminishes the role of the learner in delivering information [10, 11]. Lectures are usually delivered in a uniform manner and pace to all students although students have different learning abilities, styles, and speeds [10]. It is difficult for weaker students to follow in the teacher's steps. Therefore, not all students can absorb the content of the lecture in the same way. Thus, lecturers have limited opportunity to ask questions. Students are, therefore, forced to take notes, so that they can retrieve them later or refer to the curriculum materials throughout the session, which distracts them from the presentation of the material [8]. In addition, the quality of speech mainly depends on the experience and skills of the teacher. This is due to the teacher's environment or personal conditions [8, 12]. If the curriculum is managed in a teacher-centered way, the students will not have the opportunity to interact with the professor and learn deeply.

In addition to student dissatisfaction and feelings of lack of interaction with professors, another negative consequence of this routine classroom management is medical students' boredom and feelings of lack of connection with basic sciences courses [13]. In some cases, students are unable to choose between medical specialization and biochemistry courses as it requires them to communicate with a wide range of abstract and theoretical concepts [14, 15]. Based on principles of pedagogy, the course delivery must be adapted to students’ own needs and circumstances, as well as their speed and learning styles. Thus, students can learn in a self-directed way, gaining in-depth understanding because it is possible for them to review the course material [16]. This question is especially important because biochemistry is one of the foundation courses offered to various fields of medical sciences such as medicine, dentistry, pharmacy, nursing which usually engage a wide range of students [17] Therefore, teachers should use new and innovative approaches to enhance student learning [18, 19]. Considering the development of new technologies in this era, many studies recommend the use of new technologies in education, especially for younger generations, to integrate technology with education [20–22]. Today, most of the students are digital natives and must respond to the demands of the new digital world. For this reason, innovations in instructional processes and educational environments are necessary to meet the needs of these learners [23, 24]. The new generation of medical students are more skilled in using new technologies. Therefore, teachers should provide solutions to help students and guide them to self-directed learning based on problem solving and active participation [25, 26]. Nowadays, there are various methods of technology-based learning, and the most common term is e-learning, which is sometimes referred to as online learning. Nowadays, most educational institutions, including schools and universities, use various e-learning methods and tools, and gradually new terms such as blended learning have been added to it. To explain the dimensions of the issue, the concept of e-learning, blended learning, and its various models, as well as the models used in this research, have been described.

### E-learning

E-learning is an important type of technology-based learning, which promotes active learning. Covering a wide scope, it refers to the preparation and delivery of educational programs through electronic systems [27]. In the literature of medical education, this term is sometimes considered synonymous with online learning, computer-assisted learning (CAI), computer-based learning (CBI), Internet-based learning, learning via multimedia, and web-based learning [28, 29].

E-learning provides much more flexibility and comfort than face-to-face classes [2] as it adapts to the learner's circumstances and pace of learning by removing time and place restrictions.

E-learning allows for more practice and repeated exposure, thereby fostering deep learning in students by creating better conditions for creative learning [29–32].

Powerful interactive online tools can provide opportunities for deep learning, as some studies have shown that online learning can be as effective as face-to-face learning [33]. Meanwhile, new technologies have created enormous potential for the integration of such devices into educational settings [34].

The development of new technologies also allows to diversify student-oriented learning methods, considering the differences in learning abilities of learners to strengthen active learning in a wide range of medical fields [35–38]. In spite of its many advantages, e-learning must not be used alone in the absence of some form of face-to-face interaction. In fact, blended learning is what has been highlighted as an appropriate method for teaching and learning today, which is a new educational approach that combines face-to-face and online learning.

### Blended learning

Blended learning uses and combines the strengths of face-to-face training with new electronic technologies. Research has shown that blended learning increases the students' satisfaction with learning as learners have a greater sense of belonging due to their physical participation in class [39, 40]. In blended learning, it is possible to teach with a holistic approach and use a combination of various learning tools and methods, by considering the individual characteristics of the learner, e.g., attitudes, beliefs, views, knowledge, skills, and abilities [41, 42]. Blended learning uses a mixture of important learning strategies such as: lecture, visual and auditory demonstration, discussion, hands-on projects, with a variety of visual and audio interaction tools. Moreover, it employs collaborative learning activities and learning from peers [43]. A range of blended learning tools can be used from the simplest to the most complex, e.g., electronic message, learning management system (LMS), multimedia and educational videos, podcasts and audio files, simultaneous discussion of text, audio and video, online chat, video conference, slide sharing, and online resources for a variety of assignments and activities. Learning activities include self-testing and final assessment, forums, simulations, and virtual interactions with patients, while learners interact with teachers, peers, and course content [44].

Blended learning models are diverse. Horn and Stocker (2012) introduced various models of blended learning including self-blended or A La Carte model, enriched virtual model, flex model, and rotation models (station-rotation model, lab- rotation model, individual-rotation model, and flipped-classroom model) [45–47]. The enriched virtual model is mostly offered for virtual and full-time online courses or majors, allowing learners to complete most of the coursework online at home without the need for daily attendance, so that only a few face-to-face sessions are sufficient [46]. Self-blended courses (A La Carte model) are often used for elective courses where students can choose units or subjects according to their individual interests and conditions [45, 47]. In the Flex method, the main backbone of education is online tools such as LMS and Moodle. In this method, in addition to attending face-to-face classes according to predetermined class schedule, students interact with their teachers through an online platform and follow up learning activities [45]. Among the mentioned models, the most common and popular model is the flipped classroom method. The main point in the flipped class method is to reverse the sequence of classroom activities and home activities [46, 47]. In this research, a combination of two models (flex model & flipped classroom) was used. To explain this blended concept further, we first discussed the definitions of flipped classroom models.

#### Flipped classroom

The term "flipped class" was first coined by John Bergman and Aaron Sams, chemistry teachers at a high school in 2002, and since then this term has been developed in the field of teaching and learning and has been popular. Although initially they developed this method for students who missed or those who could not attend classes, today this approach is used in various educational areas and at different levels [48].

Flipped class model reverses the sequence of class activities, so that students receive the course content before attending the class. They go through their learning path in a self-directed way through the activities guided by the instructor, while the face-to-face class is used for research, exploration, and sharing ideas. Inspiration and motivation are accompanied by scaffolding in education and practical activities, while better quality course content is provided to students [49–52]. Hamdan et. al. believes that in the flipped classroom learning model, teachers take direct learning out of the large group learning space and with the help of modern technologies, by transforming it into an individual and individually spaced one suitable for students’ circumstances [53]. In several studies in teaching medical sciences, it has been confirmed that this method increases learning and satisfaction and improves students' performance and academic success [48, 51–57]. The flipped learning method particularly strengthens students' flexibility, active learning, and sense of responsibility as it is very effective in making better use of face-to-face classroom time [9, 34]. It potentially creates a community due to increased productivity and increases learning, interaction, and engagement with peers [9]. The course content is presented online or via educational videos to save the interaction time for teachers and students. Research has shown that the use of interactive tools does not have a negative effect on the content and students’ achievement and quality of presentation [34]. In classroom interactions, students are engaged with the videos, tests, and study work as they participate in problem-solving exercises [58, 59].

Nonetheless, features of course design are significant in the online mode. The course content and process should enjoy technical and educational quality to encourage students to get engaged in learning activities. Otherwise, there is little interaction between professors and students because students may not see their professors in person, or because of students' lack of self-regulation, or distractions in online environments, even though there is good e-content [60–63].

Recent studies have shown that students prefer educational materials produced by teachers [36]. Online video content before class has many advantages over textbooks, especially for introductory students who are new to complex subjects. Reading a textbook is primarily visual and conceptual, while video-based and multimedia instruction adds auditory appeal to visual comprehension, language comprehension, and cognitive processes, with a stronger emphasis on the importance of content. Video assignments are typically more engaging for a large introductory science course and may stimulate greater engagement with the course material [64]. Moreover, some studies have shown that providing e-content prepared before students attending the class can provide students with opportunity for practice and repetition because of time and place flexibility. Yet, in the absence of face-to-face sessions it may lead to a decrease in live interactions between the professor and the students. The flipped method attempts to solve this problem [62]. There is no single scenario for how to blend the face-to-face and e-learning classes as there are various methods, but all types of blended learning need to take advantage of both face-to-face and online methods [62–66].

### FFC model

As mentioned earlier, biochemistry is widely considered a difficult subject for both students (Learning) and teachers. (Teaching management) It requires a lot of memorization and dedication, which can be challenging for some students. Specially, biochemistry covers a wide range of topics, including metabolism, enzyme mechanisms, and genetics, which can be overwhelming for some students. Despite its difficulty, biochemistry is an important subject in medical basic sciences that is essential for many careers in the life sciences, including medicine, pharmacy, dentistry, nursing, and so on. Flipped classroom and flexible models have been found to be effective in biochemistry education. The flipped classroom model allows students to watch pre-recorded lectures before class, which frees up class time for more interactive activities such as group work, problem-solving, and discussions; also, the flex model provides students with a personalized learning experience that is tailored to their individual needs and preferences. To investigate the effectiveness of a hybrid model in teaching medical students, we decided to use a combination of the flipped classroom and flex models.

In our initial review, we found that there were few studies that have investigated blended learning methods in clinical biochemistry courses. Few efforts have been made to use educational videos in the presentation of laboratory topics in the biochemistry department of Shiraz University of Medical Sciences (SUMS) [67]. Moreover, there were few cases of using e-content and multimedia in the presentation of some parts of biochemistry education.

Since the infrastructure of the LMS existed as the main platform for e-learning in SUMS to share course contents, learning activities and formative examination, we chose the *"Flex"* approach. Hence, the contents of biochemistry topics were prepared as multimedia and provided to the students before the start of the course via the "Flipped classroom" approach. Therefore, the integrated Flex-Flipped Classroom (FFC) model was used as the intervention, with the baseline being face-to-face approach, where the usual way of presenting lessons includes lectures and discussion. We decided to compare the delivery of a clinical biochemistry course in face-to-face and FFC model.

In this study, there were four main objectives:

- 1) Comparison of students' satisfaction with the quality of course in face-to-face and FFC groups.
- 2) Comparison of students' self-evaluation of knowledge in face-to-face FFC groups.
- 3) Comparison of the students' scores of final exams (Performance) in face-to-face and FFC groups.
- 4) Investigation of students' satisfaction of course quality after intervention according to demographic variables.

## Method

### Research design

**Fig. 1:**
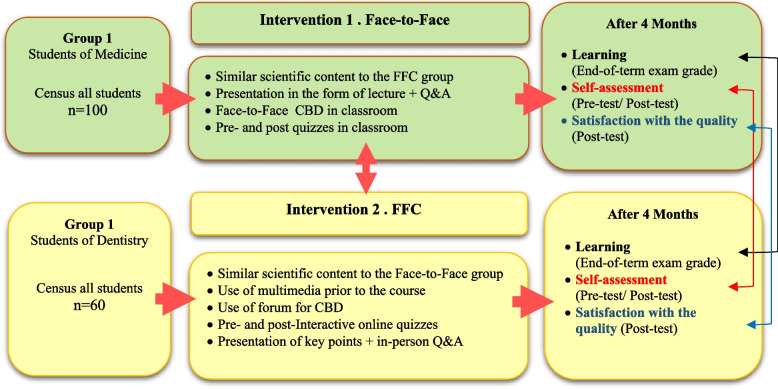
A schematic view of the research design of educational intervention in the two groups

This is a comparative quasi-experimental study employing two-group pre-posttest design, with a sample of medical and dental students in Shiraz University of Medical Sciences who were enrolled in the clinical biochemistry course in 2019. Medical students received the conventional face-to-face education, while dental students received blended learning in the form of FFC model with the standard interactive multimedia presentation via SCORM format on LMS before the classroom beginning. A variety of web-based interactive modules such as discussion forums, quizzes, assignments, and exercises were used in the experiment. The students’ satisfaction with the quality of education scores, their self-evaluation of their learning, and their final exam scores obtained on the end-of-semester test were examined in both groups after the educational intervention (Fig. 1).

### Questionnaire distribution time frame

Biochemistry course was held in the academic semester of 2018–2019 from February to July, and the questionnaires were distributed and collected at the end of the semester before the final exam in person.

### Sample size determination

To determine the minimum sample size in each group based on previous similar study [66] and using the formula below, we estimated the sufficient sample size in each group. In this formula, the mean and variance information were obtained; the students’ mean scores after the intervention were μ_1_ = 15.40, μ_2_ = 17.46, δ_1_ = 2.46, and δ_2_ = 2.36. Therefore, the minimum sample required to enter the study was 32 people.

$$n=\frac{{\left({z}_{1-\alpha /2}+{Z}_{1-\beta }\right)}^{2}\left({S}_{1}^{2}+{S}_{2}^{2}\right)}{{\left({\mu }_{1}-{\mu }_{2}\right)}^{2}}\frac{{\left(1.96+1.30\right)}^{2}\times \left(2.36+2.46\right)}{{17.46+15.40}^{2}}$$

**Fig. 2:**
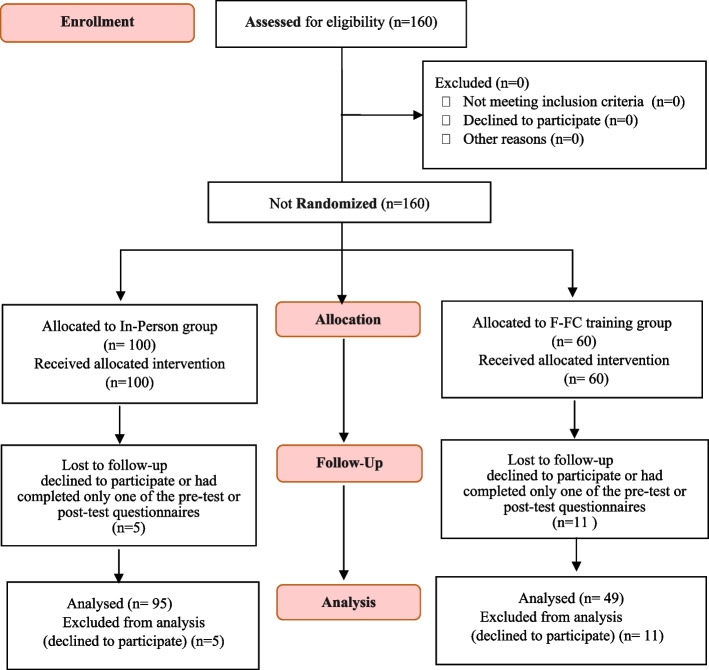
The Participants’ recruitment flow diagram

However, since all students of both medicine and dentistry were enrolled in a clinical biochemistry course in the semester of the 2018–2019 academic year (February to July), it was impossible to split the participants. We could not randomly assign students in a class to two groups due to the possibility of information leakage. Additionally, from an ethical standpoint, it was not fair to have students exposed to two different teaching methods in the same classroom as this could potentially affect the comparison of their end-of-term grades. Accordingly, we decided to select two separate groups of students who were at the same level but did not have access to each other's information (Fig. 2). The medical and dental students had similar scientific levels and had taken a similar course with the same syllabus. The course content was the same for both groups, and the instructor was also the same. Both groups used the same course material, quizzes, discussions, and final exam. Given that the students of medicine and dentistry fields are almost similar in terms of grade point average and academic characteristics, these two fields were selected for comparison. In terms of the time of presentation of the course, both groups were enrolled in this course in the same semester.

Due to the great distance between the two faculties (in two different parts of the city) and with the necessary explanations to the students of the FFC group, data dissemination between the two groups was made impossible. Both groups of students were similar in terms of academic background.

### Tools and data collection process

The research tools were two researcher-made questionnaires for measuring the students' satisfaction of the quality of the course and the self-assessment questionnaire of the participants' knowledge. In addition, clinical biochemistry final exam grades were used to compare the students' performance. Also, in a part of the questionnaire, demographic information including gender, age, GPA of the previous semester, residence status (with family, dormitory, independent house), marital status (single, married), employment status (employed, unemployed), level of access to a personal computer, and Internet access was also checked. The following are the characteristics of the research tools.

- A. Satisfaction of quality: A researcher-made questionnaire was used to measure satisfaction with the quality of the course from the students' point of view. It consists of 21 statements scored using a six-point Likert scale, ranging from strongly agree = 6, agree = 5, somewhat agree = 4, somewhat disagree = 3, disagree = 2, and strongly disagree = 1, with the cut-off line or minimum score being 3.5. This questionnaire measured the students’ satisfaction in five areas, i.e., "content effectiveness", "active learning", "questioning", "flexibility", and "general feeling towards education". After the intervention, this questionnaire was distributed among both groups, and its results were compiled.
- B. Self-evaluation of knowledge: In the self-evaluation questionnaire, some questions were asked regarding the main topics of the course, including the five main topics of the content presented during the semester, comparing the students’ knowledge before and after the course. The Likert scale questions ranged from 6 (very much) to 1 (very little). This questionnaire was used for both intervention groups.
- C. Scores of the final exam (Performance): The scores of the criterion-based summative examination (final exam) of the students in the two intervention groups (four-choice questions based on the course content) were the basis for comparing the knowledge gained by the students of the two groups. The students’ scores ranged from 0 to 20 and the minimum score to pass the course was 12.

### Validity and reliability of research tools

#### Validity

To determine the validity of the researcher-made questionnaires, at first face validity was examined using the opinion of five students and five professors. In this stage, eight statements needed to be modified in terms of grammar. Three statements were reviewed in terms of relationship with the components of the questionnaire.

Content validity was evaluated using the opinions of ten experts in the fields of biochemistry, medical education, and e-learning with Content Validity Index (CVI). Based on the content validity index provided by Waltz and Bausell [68], the experts were asked to determine the status of each item in the three areas of relevance, simplicity, and clarity with a four-part spectrum. In the end, we divided the number of experts who chose option 3 and 4 by the total number of experts. If the resulting value was less than 0.70, the item was rejected; if it was between 70 and 0.79, it was reviewed. Also, according to the number of experts (N = 10), if it was greater than 0.79, it was acceptable [68]. In reviewing the opinions of 10 educational and biochemistry experts, three questions of the index of clarity and simplicity had a score of 60%, and according to the items and explanations expressed by modifying the writing style of the items, all the items were approved with more than 80% agreement.

#### Reliability

For internal consistency test, Cronbach's alpha was used to determine the reliability of the questionnaires. All items of the questionnaires were analyzed with 45 samples. For students' satisfaction, with the quality of education questionnaire, the reliability value was 0.928, and for self-assessment of knowledge questionnaire, it was 0.934.

#### Scientific content of the course

The content of the course included the topics discussed in the table below (Table 1). All the features of the content of the two courses were the same except for the presentation method. In both groups, the clinical biochemistry topics were presented as Case-Based Discussion (CBD). In the face-to-face group, it was presented in the form of face-to-face question and answer, and in the opposite group, it was presented in the form of a discussion forum. The duration of the lessons was the same. Both groups were evaluated with the same questions and on the same day (Table 1).

**Table 1:** The content of the materials and the method provided to the intervention groups

Sub-titles	F-FC Blended Learning						Face to Face		
	Social Media	e-Quiz	Forum	Multimedia (Time)	Short Lecture	Virtual Class	Quiz	CBD	Lecture
Digestion and absorption of fats	*	*	*	40’	30’	20	5’	15’	70’
Oxidation mechanism	*	*	*	45’	30’	20	5’	15’	70’
Fatty acid biosynthesis	*	*	*	40’	30’	20	5’	15’	70’
Cholesterol metabolism	*	*	*	40’	30’	20	5’	15’	70’
Metabolism of Eicosanoids	*	*	*	40’	30’	20	5’	15’	70’

Multimedia content was prepared using Flash software and SCORM 2004 output, which included 5 main topics via standard interactive multimedia. Each content consisted of 2–3 speeches (Sco) and at the end of each speech (Sco) 5 to 7 interactive MCQ questions were presented along with interactive feedback to recall and review the material. Similarly, it was in the form of short/MCQ questions in face-to-face class. The content of the questions was similar. The research plan is displayed on the following section.

#### Data analysis

For data analysis, SPSS 24 software was used. Frequency, percentage, mean, and standard deviation were used to describe the demographic characteristics of the participants. To compare within-group pre-test and post-test scores, we used paired t-test, and independent t-test was used to compare the scores between groups in terms of knowledge, satisfaction, and performance, For comparing scores based on background variables, independent t-tests and ANOVA were used.

## Results

The clinical biochemistry course was a common course run for two groups of students including 100 medical students and 60 dental students. The content of this lesson was the same for both groups. This course was presented for medical students in a face-to-face format and for dental students in a combined FFC model.

In total, 95 medical students and 49 dental students answered the survey questionnaires completely, and all students participated in the final exam. Before analysis of the results, the overall characteristics of the two groups were first compared. As the results show, the two groups were not significantly different in terms of age composition, gender, marital status, employment status, place of residence, accessibility, GPA of the last semester, etc. (Table 2).

**Table 2:** Comparison of the demographic characteristics of face-to-face and FFC groups

Characteristics of Participants		Groups		N	Sig
		Face-to-Face (Medicine)	Blended FFC (Dentistry)		
Gender	Female	27	20	47	0.139
	Male	68	29	97	
Total		95	49	144	
Age	Mean ± St.D	19.15 ± 1.40	20.04 ± 2.41	144	0.057
Marital status	Single	93	47	140	0.270
	Married	1	2	3	
Employment status	Employed	7	5	12	0.545
	Unemployed	87	44	131	
Residency status	With Family	44	17	61	0.060
	Independent	8	1	9	
	Dormitory	43	31	74	
Access to computers (at Home)	Yes	84	37	121	0.056
	No	11	12	23	
Internet access (at home/dormitory)	Yes	92	48	140	0.054
	No	2	0	2	
Mean Score of previous semesters (Range: 0–20)	Minimum	13.70	12.40	144	0.372
	Maximum	18.85	18.88	-	
	Mean ± St.D	16.54 ± 1.23	16.32 ± 1.54	-	
	< 15	16	9	25	
	15–17	40	29	69	
	> 17	32	11	43	
Internet usage for scientific activities	0–3 Hours	88	44	132	0.769
	4–6 Hours	5	3	8	
	> 7 Hours	2	2	4	
Internet usage for entertainment	0–3 Hours	46	28	74	0.571
	4–6 Hours	22	11	33	
	> 7 Hours	26	10	36	

### Question 1

By comparing the students' satisfaction with the components of quality of clinical biochemistry education in face-to-face and blended groups after the intervention, we found that the average score of the total and sub-components was more than expected (Mean > 3.5) and significant (*P* < 0.05) as follows: “effective content” (*P* = 0.001), “questioning” (*P* = 0.006), “active learning” (*P* < 0.001), and “the feeling of the effectiveness of the course” (*P* = 013), respectively. Although both groups scored above the cut-off or minimum expected average, i.e., 3.5 out of 6, the face-to-face group scored higher than the electronic method (Table 3).

**Table 3:** Comparison of the average educational quality satisfaction in face-to-face and FFC groups

Components	Groups	Mean	Std. Deviation	t	Sig
Effective content	Face-to-Face (Medicine)	4.96	0.84	3.54	0.001
	Blended FFC (Dentistry)	4.37	1.10		
Active learning	Face-to-Face (Medicine)	4.47	1.01	3.92	0.000
	Blended FFC (Dentistry)	3.63	1.48		
Questioning	Face-to-Face (Medicine)	4.52	1.12	2.81	0.006
	Blended FFC (Dentistry)	3.92	1.31		
Flexibility	Face-to-Face (Medicine)	3.86	1.18	-1.16	0.247
	Blended FFC (Dentistry)	4.11	1.25		
Feeling	Face-to-Face (Medicine)	4.25	1.28	2.50	0.013
	Blended FFC (Dentistry)	3.65	1.50		
Total Mean	Face-to-Face (Medicine)	4.42	0.97	2.44	0.016
	Blended FFC (Dentistry)	3.94	1.24		

In the differential analysis of questionnaire items, the results showed that in most cases the average scores of the face-to-face method items were higher than those of the blended method. Nonetheless, in item 18, which refers to the possibility of adapting to the student's conditions and time constraints, the score of the FFC group was higher (4.14 ± 1.55). (Table 4).

**Table 4:** Average satisfaction of students with the quality of clinical biochemistry training in face-to-face and FFC groups

**Components**	**Items**	Face-to-Face (Medicine)		Blended FFC (Dentistry)	
		Mean	Std. D	Mean	Std. D
Effective content	1. The quality of the scientific content presented was appropriate	5.13	0.970	4.41	1.337
	2. Scientific materials were presented in order and sequence	5.11	0.916	4.47	1.138
	3. The scientific content covered the educational goals and needs	4.90	1.094	4.41	1.223
	4. The teacher gave good examples when presenting the lesson	5.02	1.097	4.61	1.115
	5. The volume and speed of presenting the lesson content was appropriate	4.82	1.154	4.24	1.562
	6. The good combination of images, text, and … was used to present the lesson	4.55	1.396	3.78	1.598
Active learning	7. There was opportunity for discussion, question and answers in class	4.06	1.343	3.45	1.672
	8. It was possible to critique, argue and think about topics	4.16	1.401	3.96	1.353
	9. 1. Students actively participated in the learning process	4.33	1.332	3.37	1.654
	10. There was ample opportunity to interact with the teacher in class	4.63	1.247	3.88	1.550
	11. I did not worry about taking notes and notes in class and focused on the lesson	4.98	1.093	3.80	1.472
	12. I learned from the questions and answers and discussions of my classmates	4.30	1.205	3.57	1.633
Questioning	13. The pre-teaching questions increased my attention to the main points of the lesson	4.31	1.383	3.35	1.866
	14. Questioning during the class after each topic, was helpful to review and memorize	4.80	1.027	4.10	1.403
	15. The short quizzes were helpful in self-assessing and identifying learning difficulties	4.48	1.143	3.82	1.629
Flexibility	16. Before the class started, I read the lesson and prepared for class	4.64	1.320	4.10	1.229
	17. After class, I had the opportunity to practice and repeat the lesson	4.47	1.354	3.86	1.414
	18. The lesson was presented was adapted the speed of my learning	3.97	1.440	4.14	1.555
	19. The presentation method was compatible with different learning styles of students	4.34	1.196	4.22	1.517
Feeling	20. I feel comfortable learning the lesson	4.16	1.401	3.96	1.353
	21. I feel more motivated to learn biochemistry	4.31	1.383	3.35	1.866

### Question 2

By comparing the students’ scores of final exams in the face-to-face and blended FFC groups after the intervention, we found that although the average achievement test score of the students in the FFC group was higher than that in the face-to-face group, this difference was not significant (*P* = 0.758) (Table 5).

**Table 5:** Comparison of students' knowledge scores of final exams in face-to-face and FFC groups

	Groups	N	Mean	Std. D	t	Sig
Scores of Summative Exam	Face-to-Face (Medicine)	100	13.65	2.30	0.30	0.758
	Blended FFC (Dentistry)	60	13.77	2.61		

### Question 3

We compared self-assessment scores of students' knowledge of clinical biochemistry course topics in the face-to-face and blended FFC groups. Having been taught five main topics of biochemistry, the students were asked to self-evaluate their knowledge before and after the course. The score range was between 1 and 6, with the cut-off point or expected mean being 3.5. The paired t-test in the last column (significant level) shows that the students considered the amount of knowledge gained in this course to be significant in all five areas. Comparison of the basic knowledge of the participants before the start of the course in the two groups showed a non-significant difference in the pre-test p value*. In other words, both groups were equal in terms of basic knowledge. Moreover, as the p value** post-test column shows, the amount of knowledge gained in both groups was not significantly different in all titles and headings. Given that the average scores were more than expected, i.e., 3.5, the results indicated that both methods were effective in improving the students' knowledge (Table 6).

**Table 6:** Comparison of the students' pre- and post-test self-evaluation in face-to-face and FFC groups

Lesson outlines	Groups	**Pre-Test**		**Post-Test**		t	Sig
		Mean	Std. D	Mean	Std. D		
Digestion and absorption of fats	Face-to-Face (Medicine)	1.66	0.945	4.09	1.457	-16.32	< 0.001
	Blended FFC (Dentistry)	1.49	0.856	3.94	1.566	-12.01	< 0.001
	Between Group Sig	*P = 0.234		**P = 0.632		-	-
Oxidation mechanism	Face-to-Face (Medicine)	1.56	0.934	4.06	1.480	-14.79	< 0.001
	Blended FFC (Dentistry)	1.41	0.762	3.78	1.403	-12.72	< 0.001
	Between Group Sig	*P = 0.239		**P = 0.262		-	-
Fatty acid biosynthesis	Face-to-Face (Medicine)	1.53	0.919	4.13	1.506	-14.97	< 0.001
	Blended FFC (Dentistry)	1.39	0.759	3.73	1.411	-12.33	< 0.001
	Between Group Sig	*P = 0.315		**P = 0.131		-	-
Cholesterol metabolism	Face-to-Face (Medicine)	1.50	0.852	4.10	1.482	-15.77	< 0.001
	Blended FFC (Dentistry)	1.43	0.890	3.84	1.477	-11.24	< 0.001
	Between Group Sig	*P = 0.570		**P = 0.322		-	-
Metabolism of Eicosanoids	Face-to-Face (Medicine)	1.52	0.974	3.81	1.771	-12.08	< 0.001
	Blended FFC (Dentistry)	1.41	1.059	3.86	1.541	-10.83	< 0.001
	Between Group Sig	*P = 0.522		**P = 0.866		-	-

*P: *P*-value of the between groups comparison in pre-test

**P: *P*-value of the between groups comparison in post-test

### Question 4

We examined the students’ satisfaction of the quality of the course and scores of final exams after the educational intervention in two groups as to the background variables.

The research population consisted of all the students of medicine and dentistry in the 2nd semester; demographic characteristics of these two groups in terms of mean age, grade point average, and year of study did not differ much. In terms of access to computers and the Internet, most had access with no comparable subgroups. However, it was possible to investigate the effect of two factors, gender, and academic average on the students' viewpoints.

### Gender

By comparing students' satisfaction with the quality of clinical biochemistry courses in face-to-face and FFC groups, we found that gender difference did not have a significant effect in the total score (*P* = 0.255) or in the sub-components (*P* > 0.05). However, from the point of view of the students in the face-to-face group, a significant difference was observed in the average quality score by gender (*P* = 0.011), with females giving a higher score to the quality of face-to-face education than males. Females also gave a higher score to "content effectiveness" (*P* = 0.006), “active learning” (*P* = 0.029), and “questioning” (*P* = 0.015) (Appendix 1). Also, by comparing the mean scores of females and males in the final exam in two groups after the intervention, we found no significant differences although the average scores of females were a bit higher than those of the males in the face-to-face education group (*P* = 0.21) and the blended learning group (*P* = 1.64).

### Academic grade point average

We divided the students' academic grade point average (GPA) into three ranges: 12 to 15, 15 to 17, and above 17. We then used the analysis of variance (ANOVA) test to compare the total average of the satisfaction scores with the quality of clinical biochemistry. However, we found no significant difference (*P* = 0.255) in the total score and in the sub-components (*P* > 0.05).

### Students’ free comments

An open-ended question at the end of the satisfaction questionnaire assessed the students' opinions about biochemistry education. We asked the participants to express their opinions about the way the course was presented. Table 7 summarizes the opinions raised by the two groups.

**Table 7:** Challenges of face-to-face and FFC methods from students’ free comments

Comments	N
FFC Group	
• I could not easily use the LMS, many times the system had problems	10
• I had trouble logging into the system and sometimes the ID and password could not be recovered	9
• The text content was not easy to see on my mobile. But the figures were useful (participants who only had a mobile)	18
• Due to the speed of the Internet, downloading files was difficult and time-consuming	18
• The contents were great and useful, but I couldn’t easily download them at the beginning of the semester	14
• The text booklet was downloadable, which was very useful for browsing at home, but downloading multimedia content was not convenient	15
• The e-contents (multimedia) were very useful, and I could repeat them regularly	25
Face-to-face Group	
• In biochemistry class, I always worry about taking the handout	8
• The professor taught very fast and sometimes I could not read the entire booklet	9
• We always recorded the teacher's voice in the class, but it takes a long time to download the voice and set the handout	8

## Discussion

One of the challenges in teaching basic medical sciences, including biochemistry, is that it takes much training time to tackle the large amount of content, the difficult structures, and specialized words. In recent years, basic science teachers have been seeking creative solutions in their classrooms by using new technologies. Thus, they are in search for more effective classroom methods. As such, they have been turning to e-learning and online methods. Nonetheless, little research has been done in this regard.

It is essential to mention in the interpretation of the results that this research was conducted before the COVID-19 pandemic. At that time, there was not much experience with e-learning and online education, and instructors often used traditional and in-person teaching methods. Therefore, students did not have much skill in working with LMS and other related technologies. As a result, a portion of the FFC method's grade was influenced by students' computer skills."

It is possible that if this study were conducted now, after the COVID-19 pandemic, the results would be significantly different. The pandemic has led to the strengthening of students' computer and electronic skills, and the skills of the instructors in utilizing electronic infrastructures have improved.

In the present study, we attempted to compare face-to-face and blended FFC methods under similar conditions of academic content and the lecturer. Examining the dimension of satisfaction, we found that the participants in face-to-face and FFC blended learning were satisfied with either method; however in the sub-components, the participants were more satisfied with the face-to-face method than FFC. This difference was especially higher in the "active learning" subcomponent, but there was no significant difference in the "flexibility" component between the two methods, where the participates rated the FFC method a bit more favorably.

Moreover, based on the results of the present research in the learning dimension, the average grades of the students at the end of the semester as well as the self-evaluation of the students did not show a significant difference between the two groups. In other words, the students in both groups had similar academic performance.

In the studies on the dimension of satisfaction, Mirzaei et al. (2012) investigated the attitude of 150 students in Yazd University of Medical Sciences towards e-learning in a cross-sectional descriptive study. Much in line with our findings, they found that the students who had experienced some sessions of biochemistry lessons in a blended learning context had a positive view towards this type of teaching method [65]. In most of the similar studies, the presence of e-content and resources are often noticed and welcomed by students in terms of the confidence and educational support it creates. As in the part of free comments of students, the face-to-face group showed that some students were worried about notetaking, while the students in the FFC training group believed that the presence of pre-prepared content would allow them to review and repeat the material. In line with our findings, Varghese et al. (2012) investigated the opinions of students about the use of e-resources of university MOOCs in a study in a medical college. In the survey conducted, 98% of the students had used the provided e-resources in different cases. Most of them found the provided e-resources useful and of high quality. Most of them used these resources to get prepared for the mid-term and final anatomical assessment in the course. The use of these resources increased steadily as the academic year progressed, and the students (83% of the respondents) stated that, due to using these resources, their comprehension had improved. Likewise, 86% of the respondents stated that their ability to answer questions in assessments had improved. Meanwhile, 73% of the respondents also said they found biochemistry interesting, and 59% stated that they felt motivated to study the subject [8].

In another study, Münch-Harrach et al. (2013) investigated the delivery of a medical biochemistry course using audio podcasts and individual study by UKE University students in the period from 2008 to 2012. They found that the students were very satisfied with this method. Podcasts were prepared on a practical biochemistry course on lipoproteins. The quality of the course was measured by indicators such as comprehensibility of content, preparation for practical parts, preparation for exams and preparation for course quizzes, and "preparation for practical parts" received the highest score [4]. Shanthikumar (2009) found that in the blended method, the use of pre-prepared podcast content was effective in enhancing the learning of medical students when presenting lectures [69]. In addition to experimental and semi-experimental studies, Birgili (2021), in a descriptive analytical study of the content of articles related to the results of the flipped class from 2012 to 2018, reported positive impact cognitive, emotional and soft skills as well as academic performance [48]. In 2021, Balakrishnan et al. also showed in a meta-analysis of 20 studies on blended learning in the field of pharmacy that the blended learning approach had a positive and significant effect on the knowledge and skills of learners [70]. Moravec et al. in 2010 showed that in large biology classes, the groups that studied the large part of the content before the class, i.e., flipped class where the time the face-to-face class was dedicated to questions and answers, quizzes, and doing homework, better grades were obtained [71].

Blended and flipped classrooms ensure that the contents of the course are available before or parallel to teaching, in addition to student educational support. Hence, they create more flexibility in terms of the possibility of reviewing the course regardless of time and place limitations. This point was confirmed both in the results of the free comments of students. However, in the review of previous research, the findings of Vaona et al. (2018) are also worth considering. In a systematic review, they found that the results and effectiveness of using e-learning depended on the research conditions. His study showed that e-learning was associated with many positive effects when compared to lack of intervention, and that e-learning showed less or similar effectiveness compared to traditional educational interventions (without access to e-learning) [72].

In a quasi-experimental pre- and post-test study on 60 medical students who had taken the biochemistry course, Jafari (2012) found that blended method increased satisfaction and motivation. He also reported that students' enthusiasm had a positive effect and led to better student–teacher communication, though overall the face-to-face method was significantly more effective [66], perhaps because the quick feedback and two-way interaction between the professor and the students created an active learning environment in the classroom. Similarly, we found that face-to-face classroom was more facilitative of active learning and discussion and questions in the classroom.

In a quasi-experimental two-group study carried out in 2015, Jensen et al. compared active flipped classroom and regular active classroom. They found that the flipped class did not necessarily lead to increased learning or a better attitude than the face-to-face class. Students performed equally well in the exams and in a final comprehensive exam. In addition, students' satisfaction with the class and achievements of scientific reasoning ability were equal in both conditions [73]. The results of this study were inconsistent with ours in terms of satisfaction, but in terms of achievement test scores, they were consistent with ours. Jensen believes that when active learning methods such as discussions and question and answer are used in the teaching methods, there will not be much difference between the flipped class and the face-to-face class, so the determining factor is the type of interaction created.

Malekigorji et al. (2020) presented the innovative model of super blended teaching, taking into account that sometimes socio-cultural differences and limited teaching time in large classes prevent students from interacting and actively learning. In this model, he presented a teaching and learning model by combining accountability system in the classroom with the flipped classroom and team-based learning. It also allowed students to use their smart devices (e.g., phones, tablets, and laptops) to answer a variety of numerical, multiple-choice, short-answer, and open-ended questions presented during classes through the CRS classroom response system to encourage them to do class activities. The Flipped-CRS approach requires students to pre-read e-learning materials and watch the recorded lectures before meetings and use their knowledge in the classroom. TurningPoint CRS software makes it possible to answer questions individually or as a team. They found that the learners positively viewed F-CRS. Moreover, the super-blended teaching and learning model increased the students’ cooperation, motivation, engagement, attendance, and academic performance, especially when using F-CRS method in teams. The ultra-blended approach enables the teachers to monitor student participation throughout the year, facilitates formative assessment, and helps teachers predict raw classroom performance in summative assessments [9]. In a study inconsistent with our findings regarding the evaluation of teaching metabolism in the biochemistry course to undergraduate students, Booth et al. (2021) found that simulation-based methods with the possibility of feedback and correction, along with a blended course, in a dynamic model and Online Computational Systems increased cognitive skills and abilities of students compared to the group that received simulation training in a non-blended learning course [74]. They concluded that in interactive teaching methods, especially those with feedback, time was a vital factor. The face-to-face classroom is facilitative of teacher's care and supervision of the students, unlike the flipped classroom method. They conclude that if there is a way to communicate and interact with the students in the flipped classroom, this method may be more effective. It should also be noted that Booth et al.'s research was conducted in 2021 and after the Corona pandemic. Considering the suddenness of the COVID-19 pandemic and its quick and binding effects on changing educational methods, after the pandemic, the students and professors became skillfull in working with electronic and computer tools, leading to a better use of blended learning and content.

However, in terms of the learning scores or academic performance, the results of our research showed that both the students' self-evaluation score and the end-of-semester score of the students in the two face-to-face and combined methods were not significantly different, which is in line with Jensen et al.'s research in 2015. [73]. In a similar study, Sajid et al. (2016) examined the effectiveness of web-based blended learning. They found that from a total of 127 students, about 22.8% felt that the professors’ lectures should be given in a face-to-face context, but almost 35% felt that one-fifth of all lectures should be given online. Students expressed satisfaction with blended learning as a new and effective learning approach. Most students reported that blended learning was useful for test preparation and concept clarification. However, the comparison of grades did not show a statistically significant increase in the academic performance of students taught through the blended learning method [75].

Rabiepour et al. (2016) reported that the post-test scores of midwifery students in the blended learning method in the fetal health assessment course were significantly better than their scores in the pre-test, indicating a significant increase. However, the post-test scores of the students of the face-to-face group were higher than the average scores of the blended learning group [76]. They concluded that the difference might be due to demographic variables, level of activity and feedback, and interaction in face-to-face classes.

However, in explaining the reason for the higher mean score of the lecture method compared to the FFC method, we can point to the way the course is presented. In our study, the e-content was prepared based on the standard multimedia principles, but the duration of each multimedia content was often close to 40 to 45 min, which caused an increase in the size of the file. Furthermore, the students complained about the low-speed Internet or the cost of downloading files. This problem might have caused limitations for students; as stated in the review of the opinions of the qualitative part of the study, the students mentioned operational problems such as low Internet speed and problems downloading files. Moreover, the type and format of the e-content file are also important in making it easier for users. For example, it is more difficult to download files with a large volume on a mobile phone, or some file formats cannot be viewed on a mobile phone (for example, EXE format files). It is important, and it would be better to consider the user's conditions. To solve this problem, it is recommended that a micro-learning approach to developing course contents should be adopted, especially in communities that face more limited network infrastructure or low Internet speed. In this regard, Prakash et al. (2017) reported that three-to-five-minute audio podcasts were accepted by students as a useful and convenient supplementary tool. They reported that students used podcasts for coursework, general reviews, and quick revision; most of them felt that podcasts helped them improve their understanding of the subject matter, clarify concepts, focus on important points, and prepare for exams. Approximately 49% of the students felt that the duration of three minutes was optimal, and the rest described the duration between three and five minutes as optimal [77].

In a study in 2017, Herbert et al. tested over 250 undergraduate students of UNSW Sydney faculties of medical sciences using the flipped teaching method in teaching pathology. The researchers changed the duration of class lectures to about 12–18 min online and designed and held the course content in the form of short courses in the form of slides, animations; highlighting the main points; and containing interactive questions and tests, and e-content. ispring software and PowerPoint were used with Scorum output. Interaction and participation and feedback in large groups were possible through an Echo360 (ALP) platform. They concluded that the flipped method and short modules increased the students' understanding and strengthened active learning. Modules were enjoyable, and this combination was effective in increasing the students' satisfaction and learning [32].

It should also be noted that although most students have access to mobile phones, not all students have access to laptops or computers. Thus, it is necessary to use the combination of image and sound more than text and sound in the creation and production of e-content. After all, on the mobile page, the writings are smaller and sometimes not readable, while the images are better seen. This point was also mentioned in the qualitative comments of the participants in this study. In a similar vein, Khojaste et al. (2022) reported that the level of students' access to electronic tools and devices affects their satisfaction with the quality of the course [78].

### Limitations of the study

The important point in the analysis of the results is that the data of this research was collected before the Covid-19 pandemic. Shiraz University of Medical Sciences is one of the most important and largest universities of medical sciences in Iran and has more than 17 main faculties and 54 research centers. In 2009, the virtual education center of the university was launched, offering basic infrastructures such as LMS and e-content development center. However, due to easy access to classrooms and students, faculty members often use the traditional and face-to-face methods, rarely using online methods in the delivery of their courses. Thus, both professors and students had very limited experience in e-learning; therefore, the computer and electronic skills of students in working with e-learning tools were limited. As mentioned in the free comments, initially a large number of students had problems of working with LMS and did not have sufficient mastery. Another limitation that future researchers need to pay attention to is that the junior students participating in this study were in the second semester, and they need to work with their professors face-to-face. It is possible that if this study were conducted on senior students, the results would be different. Also, we used two different groups of students (Medicine and Dentistry). Although we could not divide one class of students into two groups to prevent data dissemination, we tried to choose a distant field with similar initial characteristics. We suggest that in future studies, comparisons should be made randomly and from the same group of students.

## Conclusion

Both face-to-face and FFC blended learning methods were effective in enhancing the students' knowledge and satisfaction with a similar effect on their academic performance, but the students in the face-to-face group showed more satisfaction than those in the blended learning group.

It seems that factors of "interaction " in face-to-face teaching have a greater impact on students' preferences. Moreover, the students acknowledged that e-content had a better fit with their learning needs and provided greater "flexibility" in their learning process. Blended learning has the merit of advanced preparation of e-content and educational support for students, but the face-to-face method has the merit of live human interactions, lack of intermediaries, punctuality, and the direct care and supervision of students by the professor. They are especially important for new students or those with lower educational levels. In addition, it seems that various factors such as the type of interactions and classroom activities; type, volume and time of e-content; facilities and conditions of students; and type and extent of access to electronic tools and devices have an effect on the effectiveness of teaching methods. Therefore, in the educational design of courses, it is necessary to set appropriate plans according to different conditions.

## Data Availability

The datasets used and/or analyzed during the current study are available from the corresponding author on reasonable request.

## Appendix 1 Comparison of male and females’ views on quality components in face-to-face and FFC model

**Table:**

Components of Quality	Gender	Face-to-Face (Medicine)					Blended FFC (Dentistry)				
		N	Mean	Std. D	t	Sig	N	Mean	Std. D	t	Sig
Effective content	Female	26	5.34	0.55	2.83	0.006	20	4.20	1.14	-0.87	0.387
	Male	65	4.81	0.89			29	4.48	1.07		
Active learning	Female	27	4.82	0.75	2.22	0.029	20	3.23	1.57	-1.61	0.112
	Male	64	4.32	1.07			29	3.91	1.37		
Questioning	Female	24	4.93	1.01	2.47	0.015	20	3.58	1.55	-0.96	0.339
	Male	67	4.30	1.07			29	4.00	1.42		
Flexibility	Female	24	4.12	1.052	1.24	0.216	20	4.03	1.24	-0.36	0.716
	Male	66	3.72	1.22			29	4.17	1.28		
Feeling	Female	25	4.60	1.10	1.57	0.118	20	3.30	1.58	-1.38	0.174
	Male	66	4.12	1.32			29	3.89	1.41		
Total Mean	Female	18	4.91	0.76	2.61	0.011	20	3.67	1.31	-1.15	0.255
	Male	58	4.25	0.98			29	4.09	1.22		

## Abbreviations

FFC: Flex Flipped Classroom
SUMS: Shiraz University of Medical Sciences
LMS: Learning Management System
CVI: Content Validity Index
CBD: Case Based Discussion
